# Moving beyond skills acquisition: a multiple case study of situated learning in a league for children with disabilities

**DOI:** 10.3389/fspor.2023.1217349

**Published:** 2023-10-12

**Authors:** Niels N. Rossing, Sine Agergaard, Lotte S. Skrubbeltrang

**Affiliations:** ^1^ Department of Health Science and Technology, Aalborg University, Aalborg, Denmark; ^2^ Department of Social Education, University College Northern Denmark, Aalborg, Denmark

**Keywords:** handball, inclusion, community, sport, participation

## Abstract

In the last few decades, there has been a movement from individualistic and mechanistic notions of learning to approaches that turn attention to the significance of the context of learning. While these approaches have been utilized to point out the significance of the environment for skill acquisition, they have primarily been oriented towards performance-oriented milieus. Inspired by the theory of situated learning in “communities of practice” (CoP), the aim of the study is to analyze learning processes among members (participants, coaches, parents, etc.) of a diverse sporting community. The article is based on a multiple-case study of a Danish handball community named Lykkeliga (Happy League) that within a few years has attracted more than a thousand children with a remarkable diverse range of age, gender, diagnosis, and disabilities. The data collection included participant observation of training and tournament situations in two clubs over a 3-month period, along with informal interviews. The thematic analysis reveals a range of legitimate ways of participating for members of Happy League clubs, including sitting on the bench and even dating during practice. In sum, our case study sheds light on how situated learning in sporting communities may be directed towards inclusion and expansive understanding of what it means to be a sport participant.

## Introduction

Currently, many sports communities for children and youth have a dominating focus on promoting their abilities and performance (1). For instance, it has been empirically shown in a Norwegian study that competitiveness clearly structures youth sport (2). Such tendencies are not unique to sport but reflect larger societal discourses within youth culture (3). Within organized sports, children are categorized and grouped according to their age, gender, and abilities, and this is thought to facilitate their inclusion as well as ensuring fairness in sporting communities (4). Consequently, sport for children (with a range of abilities and disabilities) are often grouped according to their level of physical skills.

Such focus on abilities is also reflected in the field of learning and skill acquisition in sports, where researchers and practitioners are directed toward overcoming constraints in training so athletes can acquire skills (e.g., technical and tactical) (5). The primary perspective on learning as skills acquisition is linked with a traditional notion of sport participation, which assumes that “participants follow directions and are expected to execute the skills taught and trained as needed to compete” (6). While this focus on learning may lead to sport-specific skill acquisition among athletes, it does not contribute to our understanding of how sports communities can facilitate learning among groups and individuals that tend not to participate in sports.

It is vital to understand what may facilitate not only access to sports communities, but the more continuous process of learning to become a participant in sport communities (7). This is a highly relevant topic since there is a great disparity in the degree to which and ways in which children participate in sport. For example, studies in the United States, Australia, the United Kingdom, and Denmark (8); (e.g., 9) have identified that children with disabilities participate at lower rates than the general population in all forms of cultural life, including sports (e.g. (10), Further, there are several studies indicating that the sport participation of children and youth with disabilities is often restricted with less enjoyment (11), less variety (12) and less likelihood of engaging in skill-based activities (13). In fact, accumulating evidence suggests that a range of environmental barriers such as institutional (i.e., clubs refusing to include athletes), social (i.e., labelling children negatively), and lack of support (i.e., too few staff and service providers) are some of the key barriers that restrict sport participation among children with disabilities (14–16). Thus, how sport communities organize and deliver sport is key to sport participation among children with and without disabilities.

This article aims to expand the current focus on learning as skills acquisition in sport through analyzing how sport communities may facilitate learning of what it means to be a sport participant. To do so, we study the case of the Danish Handball initiative “Happy League” that is frequented by children with diverse disabilities. Utilizing Etienne Wenger's theory about situated learning in communities of practice (CoP), we will analyze and discuss the variety of ways in which children with disabilities learn what it means to be a participant in the Happy League sporting community. In so doing, we seek to contribute to shift the focus from skill acquisition to situated learning, pointing to the options for members in inclusive sport communities to acquire experiences with diverse forms of sports participation.

## State of the art

In the last few decades, there has been a movement away from individualistic and mechanistic notions of learning within the sport sciences. While coaches have previously been meant to provide “pre-programmed optimal movement solutions” for athletes, the focus of practitioners and researchers has turned to more ecological approaches in which sporting practitioners are viewed as “sporting ecology designers” (5). Based on theories such as “ecology dynamics”, researchers and practitioners have broadened the notions and practices of skill acquisition considerably by including attention to features such as the socio-cultural norms of the sporting milieu (17). While ecological dynamics have added more contextual perspectives to skill acquisition, the central tenet of the line of research is to consider how athletes can acquire sport-specific skills, and therefore how to design training and practice. For instance, through small case studies with an elite Australian Football League (AFL) team, Pinder and colleagues (18) showed how coaches can manipulate informational constraints within drills to direct athlete's attention to relevant affordances. From an ecological dynamic perspective, learning is viewed as “an ongoing dynamic process involving a search for and stabilization of specific, functional movement patterns” across the performance landscape as each individual adapts to a variety of changing constraints (19). While these contextual-oriented theories such as ecological dynamics have offered a nuanced understanding of how athletes can acquire skills, it seems to take athletes' *participation* in sport activities and their contexts for granted as it focuses on learning of specific “functional movement patterns” from a performance-oriented perspective. Thus, with such approaches, researchers who examine learning in sport may unintendedly strengthen the attention on how to facilitate sport performance in youth sport instead of how to facilitate participation in sport in general.

In line with calls from several researchers (20, 21), we argue that there is a need to turn toward learning theories that encompass an understanding of learning as structured around participation within sport communities. Grounded in the theory of situated learning in “communities of practice” (CoP), participation means more than simple physical engagement in certain activities, as participation is also the “process of being active participants in the *practices* of social communities and constructing *identities* in relation to these communities” (22). As such, participating in CoPs involves “…doing in a historical and social context that gives structure and meaning to what we do” [28, p. 47]. Consequently, we need to expand the conventional understanding of learning as skills acquisition (e.g., executing drills) to consider also how learning in sport communities may involve acquiring the meaning of sport participation.

There is a great potential in understanding how athletes are embedded in a larger social-relational context such as a CoP and how their skill acquisition may involve learning what it means to be a sport participant. As suggested, identities among sports participants are also acquired in CoPs that “can serve as conduits not only for learning, but also for transforming sport cultures into entities primarily concerned with developing athletes” (20). Consequently, the development of athletes involves not only performance and personal development but also participation in the sport (23). Based on an ecological framework, Cote and colleagues suggest that sport participation can have short- and long-term benefits for children's competencies, confidence, character, and connection. While these certainly are fruitful personal assets, they are highly attached to the situated learning in specific practices that children encounter. Until now, most studies utilizing a CoP approach have been designed as single case studies of specific sport clubs to reveal how situated learning occurs and what (adult) athletes learn explicitly and implicitly (24–26). However, several researchers have also examined how CoPs facilitated among coaches within and across clubs could enhance their learning individually and collectively (20, 26, 27). To our knowledge, no studies have investigated situated learning among exceptionally diverse youth athletes across clubs, teams, or institutions based on a CoP approach. Further, studies with a CoP approach is often focusing on relatively homogenous groups that are similar in terms of age, gender, and abilities. Thus, in former studies of sport-related CoPs, the span in participation trajectories for newcomers or less abled members have been somewhat restricted, and the negotiation of meaning along with the construction of identities among members of the group have appeared alike.

## Theory

CoP is framed as a social learning theory; thus, learning involves social participation. The members of a community of practice will share and deepen their knowledge and expertise by interacting with one another on an ongoing basis (28). There are certain characteristics that define a community of practice: *mutual engagement, joint enterprise,* and *shared repertoire* (22). In a handball community, this could be a team training together (*mutual engagement*), wearing a club uniform (*shared repertoire*), and participating in sport towards a common goal (*joint enterprise*).

One of the key concepts in this theory is *participation*. Participation is both actions and a form of belonging. Thus, participation shapes not only what we do, but also who we are and how we interpret what we do (22). Social participation is therefore both a process of learning and of knowing that can be constituted in four components. The components comprise meaning, practice, community, and identity, which are deeply interconnected and mutually defining. Meaning can be learning as experience and is defined as our ongoing capability to experience our life and the world as meaningful. Practice is learning as doing as it is historical and social resources and our perspectives that can sustain mutual engagement in action. Moreover, community is learning as belonging which points to the fact that our participation is recognizable as competence. Lastly, learning is becoming as learning transforms who we are and creates identity.

In line with other learning theories, CoP assumes that only through participating can one learn from the given community of practice. However, contrary to other learning theories, Wenger developed a rich account of how people learn through participation in the community practices, and therefore suggests several categories ranging from participation to non-participation: *core group* (often a small group of persons whose engagement nurtures the community), *active participants* (members whose activity is recognized as significant and highly influence define the community), *occasional participants* (this group only participates when it is of special interest or they have something specific to contribute), *peripheral participants* (this group has a continuous connection to the community, but they have less commitment or authority within the community and are identified to be newcomers), and *outsiders* (persons who are categorized as not part of the community) (28).

As participation may be legitimate while also peripheral, Lave and Wenger (29, p. 36) acknowledge that there are “multiple, varied, more or less engaged and inclusive ways of being located in the field of participation defined by a community”. Based on this theoretical perspective, the findings from a physical education setting show how students' participation or non-participation are closely related to the legitimacy ascribed to them by the environment (e.g., peers and teachers), along with the level of meaningfulness experienced by the students (30). Thus, each athlete's participation does not only depend on their perceived abilities but also relates to how peers and teachers interact.

## Material and methods

The ontological position in this research project was grounded in neo-pragmatism, as we sought to produce practical truths as those that prove useful within specific contexts (31). Therefore, this study does not seek to uncover reality, but to explore habits of action for coping with reality (32). In this project, the habits of action refer to the specific practices on and off the court that we were able to observe during our fieldwork (e.g., competitors applauding each other, players doing all kinds of things during practice, coaches using concrete artifacts in practice, etc.). As Richard Rorty (33, p. 173) proposed, the way to re-enchant the world is to stick to the concrete. Knowledge is not simply a matter of representing the world accurately but of guiding effective action. Accordingly, research may serve to generate novel descriptions of a topic or context to best position others to benefit from that information practically (32). In relation to our epistemology, we recognize that knowledge construction is contextual and inherently influenced by cultural, political, and historical conditions. As such, we will describe the broader context of the case we explore in the following sections.

The present paper is based on a quintain multiple-case study (34) as this study analyses a new Danish handball initiative called Lykkeliga (Happy League). A quintain is something that we want to understand more thoroughly, and we choose to study it through its cases by means of a multiple-case study (34). In line with a quintain approach, we acknowledge that Happy League is a target of our investigation but not a bull's eye as our fieldwork (besides official tournaments) only comprises two of the 78 club teams. Although each case (club team) was interesting to us, we focused on cases that could reveal embedded information about the quintain (Happy League).

Happy League was initiated in 2017 and since then has sparked the participation of approximately 1,200 new handball players with disabilities in 78 new club teams nationwide in Denmark. Happy League welcomes youth across age, gender, and abilities who do not feel they belong in the mainstream sport clubs. One of the few written core espoused values of Happy League is that the community is about much more than handball and that they encourage positive thinking and togetherness. The teams in Happy League are organized within traditional sport clubs and are widely dispersed geographically, including teams on the Faroe Islands and in Greenland. Happy League has used social media (Facebook, Instagram, and TikTok) to share their stories about their national and local initiatives with such success that Facebook visited them in 2020 and posted a video about their community.

The data collection was based on field studies in two clubs over a 4-month period in one club and a 6-month period in the second club due to COVID-19 restrictions, along with 15 interviews with parents within seven clubs. In this article, we focus solely on the material generated during the field studies that were carried out in two clubs located in two different regions of Denmark.

Two local head coaches acted as gatekeepers who granted access for the first author and the research assistant to conduct field work in their clubs. During the field work period, the first author and a research assistant conducted observations of weekly handball sessions at two separate sites along with interviews with club officials and regular participants. At both sites, the Happy League teams were situated in traditional recreational clubs with teams ranging from Under 6 to adult teams.

Studies of children's sporting development (35) and, in particular, studies among children with disabilities (36, 37) clearly state that we easily consider children as our object of research, and therefore the voices of children are missing. Consequently, we were specifically interested in doing participant observation to learn from the children, as *“…to observe with or from is not to objectify; it is to attend to persons and things, to learn from them, and to follow in precept and practice”* (38, p. 61). As the children we followed had various disabilities, participant observation was the most suitable method to somewhat approach the children's' voices, as several of the children observed had limited or no verbal language. While our observations of children did not make us capable of replicating the exact words that the observed children said, it helped us understand what was meaningful for the children. As listening to children's voices can be defined as an active process of exchange of meanings (39), the participant observation was crucial to approach the children's perspective as near as possible.

Using “participant as observer” positions (40), we took on various roles during training; we instructed handball drills, fooled around with the players in the small breaks, had informal talks with parents and coaches, and took part in the games at the end of training as active coaches. Further, we were observers in several tournaments as this was an important part of the routines within the teams. This provided us with unique opportunities to engage in informal conversations, for example about participants' (both parents and players) reasons for engaging in Happy League, their participation trajectories, and to follow new players' inclusion into the team. Field notes were drafted during training and tournament sessions and written more extensively after field work, as suggested by Sparkes and Smith (40) along with Buch and Staller. Written informed consent was obtained from the players' legal guardian/next of kin. To ensure the anonymity of the interviewees, we removed all identifying information and assigned pseudonyms for each participant.

### Analytic strategy

We employed a reflexive thematic analysis in which the “researcher's reflective and thoughtful engagement with their data […] and the analytic process” was central in analyzing our field notes (41, 42). Inspired by Clarke and Braun (41), the first author reviewed the field notes prior to proceeding with an inductively data-driven approach, in which similar segments of text were first identified and grouped. Further, turning to a more deductive process, the first author reread the field notes and the segments of the text to possibly link the segments chosen to specific theoretical terms from the communities of practice approach. For instance, this process led to the theme *Learning through artifacts* as it is closely linked to the theoretical term reification. Hence, the first author organized the segments into broader and more manageable themes. These themes were carefully reviewed, discussed, and refined as a research team, which resulted in three themes.

To ensure qualitative rigor throughout this case study, we established and followed certain procedures. To establish credibility, the concept of crystallization was used to provide a complex, in-depth, and thorough understanding of this specific case study (43). Two researchers conducted field work to shift between multiple and consistent researcher viewpoints throughout the process. Peer-debriefing was used after each observation to purposefully reflect on their own preconceptions and ongoing interpretations.

The third author, who had intimate familiarity with the community due to another research project, assisted with member reflection during the observation period and in the period of analysis. Also, a member reflection between the first and third author and a leading person within Happy League was accomplished before the analysis was determined. Member reflection was aligned with the reflexive thematic analysis to provide an “intellectually enriched understanding through generating additional insights and dialogue” (44). Lastly, a leading figure of Happy League and the co-authors in this study also engaged as critical friends to facilitate rigor and quality through critical dialogue (44). The ongoing dialogues functioned as reflexive elaboration, ensuring that our interpretations of the data were challenged, and provided an opportunity to reflect upon alternative explanations or perspectives to enhance the quality of this case study. We now present the results of our analysis.

## Results and discussion

The following analysis is split into three parts. Initially, we point out how centrally designed artifacts within the community facilitated *Learning through artifacts*, which helped athletes and parents to learn sport-specific skills and unite athletes, parents, and teams. Secondly, we focus on how coaches, parents, and athletes were *Learning to be inclusive* on and off the court. Thirdly, we reveal how the community enabled practices so all involved were *Learning that sport participation comes in many forms*.

### Learning through artifacts

Already in the first visits to the clubs, we noted that Happy League fosters a shared repertoire across coaches, parents, and players by promoting appealing distinct physical requisites. For instance, an identical Happy League logo was used on various jerseys across the club teams and was something that was striking for the author from the very first observation day. During our field work, we realized that these symbolic artifacts not only functioned as a shared repertoire, but that the members of the community were learning an extended notion of sport participation through artifacts. During an informal interview with a local coach, it became clear, that the Happy League logo reinforced to members (e.g., athletes, coaches, and parents) that they are part of a larger community across gender, age, disability, clubs, and even countries.

When we went to the Happy League tournament last time, we were a large group of people and went there the day before to be properly ready for such a big day. Since we came early, we went for a stroll down a pedestrian street. At some point, we saw a small group of people going directly toward us. Within the group, this one guy with a Happy League shirt smiles and starts to hug us. All. He was from the Faroe Isles, and was not able to speak, but was so glad to meet other Happy League people, which he could figure out we were as we, of course, also wore our own Happy League shirts. (Field notes from first author, 31/1–2022)

Through the community-designed artifacts, members of the club teams non-formally learn that they are not only competitors, but are also embedded in a larger community with similar interests such as having fun and being together. From a theoretical point of view, concrete artifacts are a way to facilitate reification, which means to project our meaning into the world through objects such as a logo (Happy League logo). As Wenger points out, reification and participation enrich other (45) and function in a dialectical relationship. While the mascot-like artifacts seem to be positive inputs occasionally, the logo is an integral part of the everyday life of the members of the group. Some of the athletes wore their shirts with the logos all the time (i.e., at school, home, and during sport) as this probably presents an important part of their identity. Thus, the logo also facilitates their participation on as well as off the court. Contrary to the previous studies utilizing the CoP framework (26, 46), the community-designed artifacts in this case play a significant role in symbolizing this embeddedness, and as shown in the observation, function as a way of connecting members within but also across club teams. The concrete community-designed artifacts carry varied functions, as a mascot named Happy-Lars mostly spreads a happy atmosphere at tournaments but also tours across club teams to spread joy to the children.

The community-designed artifacts are also highlighted within a Happy League initiative in which all players are selected to the “biggest national team in the world”. Practically, this means that each athlete has been officially selected to the national team of Happy League, symbolized in them receiving a national Happy League shirt and invitation to monthly regional national team practices across club teams. Thus, several players in one of the club teams wore national team jerseys, went to the national team practices together, and also felt a certain belonging to this community.

Before the training session begins, a coach walks over to me and introduces himself. Jarvis is the name. While we chat, a player named Joe, walks over to Jarvis and shakes his hand. “Welcome to the club, Jarvis. I am the captain for the team,” Joe says, while he first points at the armband and then the logo of the club on his shirt. “Well, that's good to know, Joe,” Jarvis replies. Joe pulls up his shirt and shows of his national team jersey and says, “And I am also captain for the national team.” (Field notes from first author, 14/2–2022)

The national team seems to serve as an artifact symbolizing that all members within the Happy League community have equal access to participation. In this sense, the community transcends the notions of a traditional national team, selecting those with the highest abilities. Furthermore, through such practices, members of the community learn that they are legitimate members of the community no matter their level of abilities and disabilities. Such an apparently unlimited inclusive approach in the Happy League community will be described in more details below.

### Learning to be inclusive

While the training directly focused on sport-specific competencies, we learnt through observations and informal interviews with coaches, parents, and Happy League staff that participants within the community informally learned to be inclusive. While all the members were deliberately focused on the children's abilities to learn to catch and throw the ball in the training activities, learning to be inclusive certainly seemed to take place more informally, though the inclusive approach was still omnipresent in all their activities across members (e.g., coaches, parents, and players). Using Wenger's terminology, the analysis suggests that learning to be inclusive is a vital part of what can be called the CoP's joint enterprise. During our field work in two Happy League clubs, it became quite evident through observations and informal interviews that their joint enterprise could be understood as creating a fun and inclusive handball community. As such, the participants' legitimacy is not linked to how they perform or even participate in the activity but simply with the fact that they join in the activity in some way. Seeing that the goal was to create an inclusive handball community, the way activities were structured taught children how to be inclusive. This was evident at both training and tournaments, and on and off court. The inclusive approach was emphasized, which showed a high degree of non-formal learning within the activities on and off court. During training, the players learned to adapt their passes and revise the rules within the handball game to ensure that all players could participate:

When a playful warm-up is over, there are multiple players who say “cue ball” or “aren’t we gonna play cue ball”. “Ok,” the head coach says. She starts cue ball with all coaches and players… Large and small (players) are part of the game. The large hard-shooting boys shoot with full power, but never against the small ones. There is an understanding of who the boys can shoot full power at, and who to shoot slowly at. At a time, the youngest takes ten steps, and starts counting on the seventh step, one, two, three. It does not create conflict. The game is on. (Field notes from research assistant, 3/2–2022).

Since Happy League invites all players that do not feel they belong in traditional clubs, they have players with high handball competencies and no labelled diagnosis on the same team with players that sit in a wheelchair or have difficulty running, walking, or catching a ball. As such, coaches informally facilitate that members of the community learn to include every teammate and even opponents in the community established around training and match practices.

In the matches during training, the coaches, including myself, act as a kind of playing coach. There are playing coaches on each team that all ensure to bend the rules according to the players' individual needs and skills. The coaches defend extra hard against Nathan and rule ordinarily (e.g., maximum three steps) when he has the ball. Mason stands a little confused on the pitch in his first training and seems to observe the coaches' ways of regulating the game. Mason takes four, five, six steps and throws the ball toward the goal and scores, yet the coach does not rule against him. Nathan asks the coach: “Wasn’t it a foul?” The coach replies: “There are no fouls on Mason today, but you only have three steps, Nathan”. Nathan looks up, and nods accepting, but also looks a bit frustrated. (Field notes from first author, 29/11–2021)

Our observations show that the practices within Happy League constitute how players learned personal competencies such as empathy *because* of the diverse heterogenous group of players within the community.

 “He has learned to be really good at taking the youngest into consideration when he plays, adapting his passes, taking good care that everyone is included. So, the fact that the athletes have various ages and gender does not stand in the way for him?” I ask. “No not all, on the contrary,” the dad says. (Field notes from research assistant, 17/11–2021)

While the coaches certainly were guiding how to be inclusive within the club teams, the parents and even the children also constituted such notions and practices during training session.

The head coach has not been at training for a long time, and she tells the athletes how much she has looked forward to seeing them. Maybe she can't remember all of their names, she says while she smiles cheekily. “We better start with a round of names”, one of the big boys says returning the coach's cheeky smile. They start to do a round of names. “The adults, too,” one of the boys says, when the head coach intentionally misses one of the parents. All, including the parents, present themselves while they look into each other's eyes, and then they are ready to play handball. (Field notes from research assistant, 3/2–2022)

Such practices were also evident in the largest tournament within the community, Happy Cup, as players had to register themselves as participants, which led to teams being formed for the tournament across the traditional club teams. This provided many players with the opportunity to play and get to know various players from other club teams, but also to learn to meet new people guided by coaches and parents. Thus, within this community, it seems that the coaches not only design drills that enable players to learn handball-specific drills, but the coaches and parents also co-create a community in which they enable players to learn to be more inclusive towards the vast diversity of players within the community. The inclusive approach was clearly not just a personal asset that the children learned (as well as coaches and parents), but also positively influenced the unity within and across teams and the participation trajectories of especially the newcomers as they rapidly felt welcomed and part of the community. These findings are similar to those found in a study among adult swing dancers, since the dancers quite freely pointed out in interviews that swing dancing was a “great opportunity for social levelling” (46). Similarly to handball, swing dance also encompasses competitive activities, central members within the swing dance community emphasize that swing dancing is not a sport *per se*, but rather an opportunity to be together and have fun. This suggests that despite the ingrained, competitive nature of sports, the emphasis from central members within a community may have the potential to inspire newcomers' meaning-making of their engagement in the sport.

### Learning that sport participation comes in many forms

Finally, the analysis reveals that members of Happy League learn that sport participation can take many forms. During our field study in both clubs, it was accepted by coaches and parents that players went for a break in the lobby, sat on the bench for the whole training, or dribbled and played for themselves during drills. This again, relates to the joint enterprise. As we wrote in the previous section, joint enterprise could be seen as creating a fun and inclusive handball initiative; meaningful participation is linked to joyful and legitimate participation, and not with winning/losing and/or skill acquisition. The coaches allow for many new opportunities for legitimate participations that in a different CoP might have led to exclusion instead. The following extract from a field note shows the legitimacy in the various ways of participating during training:

During the classic warm-up, Kayden walks over to and hugs me tightly, a real bear hug, with both his arms swung around me while he cheekily laughs. Previously, we have only said “hey” to one another. After a long firm hug, Kayden starts to wrestle with me by laying his arms around me in a firm position. Kayden cheekily smiles (he hasn't got much verbal language), while he wrestles me to the floor… The assistant coach, Jarvis, walks over to us, and I expect that he will ask us firmly to stop. Instead, he asks “Are you ok?” I look bewildered at him and says, “Yes, of course”. Kayden laughs and pulls me down on the floor again, while he holds my hands firmly to the floor. Suddenly, a girl, Cathryn, who is wearing a dressed-up outfit, not handball clothes, walks over to us. She asks quietly if Kayden wants to go outside. Kayden says “yes” while smiling. They walk quietly outside the arena, while players and coaches are focusing on drills. After five minutes, they enter the arena again hand in hand, and immediately Kayden runs over to me. I ask Kayden, “What have you two been up to?” Kayden answers that Cathryn has given him a boyfriend/girlfriend present. I answer that it sounds wonderful, while I can see his girlfriend walk towards the goal in her dressed-up outfit to participate in a new drill. (Field notes from first author, 06/12–2021)

Indeed, our observations show how all players were considered *legitimate participants* no matter what they were “practicing”. Although previous studies within disability sports have problematized that people with disabilities are framed as *viewers* rather than *doers* in recreational and sports settings (47, 48), our observations suggest that such roles often function as starting points for an *active participation* eventually*,* and also function as a pause from sport when needed. Such positioning in what might be considered peripheral roles appears to be legitimate way of participating in the handball community that is accepted by coaches along with parents. For instance, although Cathryn could be labelled as an *outsider* and a *viewer* in the beginning of the above mentioned training session, she had a meaningful non-sport task to complete before she took up the position as an *insider* of an organized sport community. Thus, by making it legitimate for participants to take up a variety of unconventional roles as sport participants that do not involve being physically active all the time, the Happy League community seems to constitute trajectories for the athletes toward sport participation.

Karl is really happy to play in (the club team), the dad explains. “There's room for all kind of children. We have tried with swimming in the local club, but we were kicked out,” the dad says. “We offered to be in the water with Karl, but that wasn’t an option, so Karl couldn’t stay on the team. Karl needs adult support. He gets that here, and at the same time there is room for him and a lot of other different children,” the dad says. (Field notes from research assistant, 08/12–2021)

During tournament games, we have also witnessed players taking the roles as judges, eating a hotdog while being in defense, chatting with opponents, taking a role as an assistant judge, while both parents, coaches, and judges were acknowledging their various forms of participation. These occurrences also clearly show that the competitive nature of the matches have been downscaled considerably compared with other sport communities (20), but also that the sport community legitimize various ways of participating as the coaches are not rewarded according to their win–loss records, but rather their way of including various players and creating sustained sport participation among them.

## Conclusion, limitations, and recommendations

In this article, we have shown how it is possible to shift the narrow focus on skill acquisition in youth sport toward situated learning, highlighting the possibilities for members in inclusive sport communities to acquire experiences with diverse forms of sports participation. As we highlight in our state of the art, many studies have either taken athletes' participation in sport activities and the meaning of the context for granted because the main objective has been driven by a performance-oriented focus or, if inspired by a CoP-approach, the studies have been on homogenous groups with little to no attention paid towards participation trajectories for newcomers or disadvantaged members. Based on Wenger's concepts of situated learning within communities of practice, this case study broadens our conventional notions of sport participation by showing that participation can take many forms, even in a highly heterogenic group of young athletes.

In our analysis, we identified three central components that made it possible for the participants to learn in various ways within the community. We argue that centrally designed artifacts facilitate the participants learning as they aid athletes and parents to learn sport-specific skills while also uniting athletes, parents, and even club teams across the countries. In our analysis, we also showed how sport participation can encompass non-physical activities during training or skill training outside the handball arena. The findings show how the players learn to play handball in a community in which competition is not ubiquitous. On the contrary, because the focus was on participation and *not* performance, players were able to learn to be inclusive while competing.

While Happy League is an inclusive sport initiative aimed at children and youth with disabilities, the approach that they have to learning and participation is not limited toward disability initiatives. The focus on competition, performance, or even enhancing sport-specific skills in youth sport in general, possibly restricts children and youth from starting participation or even dropping out at early stages. Thus, the approach to sport participation that we have presented using the case of Happy League has the potential to inform and inspire other sport systems that may be single-mindedly focused on skill acquisition, athletes' abilities, competition, and doing sports physically while they forget to nurture athletes' identity, their relationships, and the community encompassing the athletes.

## Data Availability

The raw data supporting the conclusions of this article will be made available by the authors, without undue reservation.
